# YouTube Videos as a Source of Information About Clinical Trials: Observational Study

**DOI:** 10.2196/10060

**Published:** 2018-06-26

**Authors:** Grace Clarke Hillyer, Sarah A MacLean, Melissa Beauchemin, Corey H Basch, Karen M Schmitt, Leslie Segall, Moshe Kelsen, Frances L Brogan, Gary K Schwartz

**Affiliations:** ^1^ Department of Epidemiology Mailman School of Public Health Columbia University New York, NY United States; ^2^ Herbert Irving Comprehensive Cancer Center New York, NY United States; ^3^ New York Presbyterian Hospital New York, NY United States; ^4^ Columbia University School of Nursing New York, NY United States; ^5^ Department of Public Health William Paterson University Paterson, NJ United States; ^6^ Division of Hematology and Oncology Department of Medicine Columbia University Medical Center New York, NY United States

**Keywords:** clinical trial, cancer clinical trial, social media, internet, YouTube videos, health information

## Abstract

**Background:**

Clinical trials are essential to the advancement of cancer treatment but fewer than 5% of adult cancer patients enroll in a trial. A commonly cited barrier to participation is the lack of understanding about clinical trials.

**Objective:**

Since the internet is a popular source of health-related information and YouTube is the second most visited website in the world, we examined the content of the top 115 YouTube videos about clinical trials to evaluate clinical trial information available through this medium.

**Methods:**

YouTube videos posted prior to March 2017 were searched using selected keywords. A snowballing technique was used to identify videos wherein sequential screening of the autofill search results for each set of keywords was conducted. Video characteristics (eg, number of views and video length) were recorded. The content was broadly grouped as related to purpose, phases, design, safety and ethics, and participant considerations. Stepwise multivariable logistic regression analysis was conducted to assess associations between video type (cancer vs noncancer) and video characteristics and content.

**Results:**

In total, 115 videos were reviewed. Of these, 46/115 (40.0%) were cancer clinical trials videos and 69/115 (60.0%) were noncancer/general clinical trial videos. Most videos were created by health care organizations/cancer centers (34/115, 29.6%), were oriented toward patients (67/115, 58.3%) and the general public (68/115, 59.1%), and were informational (79/115, 68.7%); altruism was a common theme (31/115, 27.0%). Compared with noncancer videos, cancer clinical trials videos more frequently used an affective communication style and mentioned the benefits of participation. Cancer clinical trial videos were also much more likely to raise the issue of costs associated with participation (odds ratio [OR] 5.93, 95% CI 1.15-29.46) and advise patients to communicate with their physician about cancer clinical trials (OR 4.94, 95% CI 1.39-17.56).

**Conclusions:**

Collectively, YouTube clinical trial videos provided information on many aspects of trials; however, individual videos tended to focus on selected topics with varying levels of detail. Cancer clinical trial videos were more emotional in style and positive in tone and provided information on the important topics of cost and communication. Patients are encouraged to verify and supplement YouTube video information in consultations with their health care professionals to obtain a full and accurate picture of cancer clinical trials to make an adequately informed decision about participation.

## Introduction

The release of new cancer treatments to market has outpaced all other therapeutic areas [1], with the introduction of 70 oncology treatments for more than 20 different tumor types over the past 5 years alone [2]. Bringing a new cancer treatment to the clinical setting is a complex process that extends over many years from the initial discovery and development in the laboratory through Food and Drug Administration (FDA) approval for use [3]. Integral to this process are the heavily FDA-regulated clinical trials that rigorously determine the safety and effectiveness of new and promising treatments among humans in an experimental setting [4].

Clinical trials are designed to answer specific research questions and are, thus, governed by strict protocols and eligibility criteria. Informed consent documents, which provide potential participants with detailed information about the purpose of the study, procedures to be performed, potential harms and benefits of the experimental agents used, and the voluntary nature of participation, are federally mandated to ensure that participants make informed decisions regarding enrollment. Much of the information presented to a patient is complex, incorporating translational research, biomarker selection, and sophisticated study designs into trials [5], but the level of health literacy remains low in the adult US population [6]. Fewer than 5% of adult cancer patients enroll in clinical trials [7], the most commonly cited barriers being lack of awareness or knowledge regarding clinical trials [8-14] and the reluctance to be randomized [15-19].

In one study, 92% of cancer patients reported the internet as the resource that empowered them when making treatment decisions and provided them with information with which to engage their physicians in discussion [20]. Cancer patients also use the internet to seek clarification, more detailed information, or reassurance about what was learned after a clinical encounter about clinical trials [21,22]. Much of the clinical trial information available online has been characterized as variable in quality with poor readability [23]. While clinical trial search tools are relatively easy to locate on the internet using various search engines, both content and functionality were also highly variable, and users needed a fair amount of knowledge about their condition and good web navigation skills to access the relevant information [24]. In a study that simulated the search for treatments of four common cancers by naïve cancer patients without clinical trial knowledge, only 85% of cancer-treatment sites mentioned clinical trials on the landing page and only 68% provided links to trials [23]. Another study that evaluated the navigability of comprehensive cancer center websites to clinical trial information observed that clinical trial content is narrow in scope with trial descriptions written at a college reading level, thereby limiting understanding for the average user [25]. When the written word proves to be challenging, consumers may turn to video-based information.

First introduced in 2005, YouTube is the second most visited website worldwide, and it has become an increasingly important medium through which health information is exchanged between and shared by consumers and health care professionals, government and nongovernment agencies, and industries [26]. Recent statistics indicate that currently, 300 hours of videos are uploaded to YouTube every minute and almost 5 billion videos are watched by 30 million visitors every day [27]. Despite the extensive reach and pervasive use of YouTube videos, little is known about videos related to cancer clinical trials; thus, the aim of this study was to evaluate the content of the most widely viewed YouTube videos related to clinical and cancer clinical trials.

## Methods

**Figure 1:**
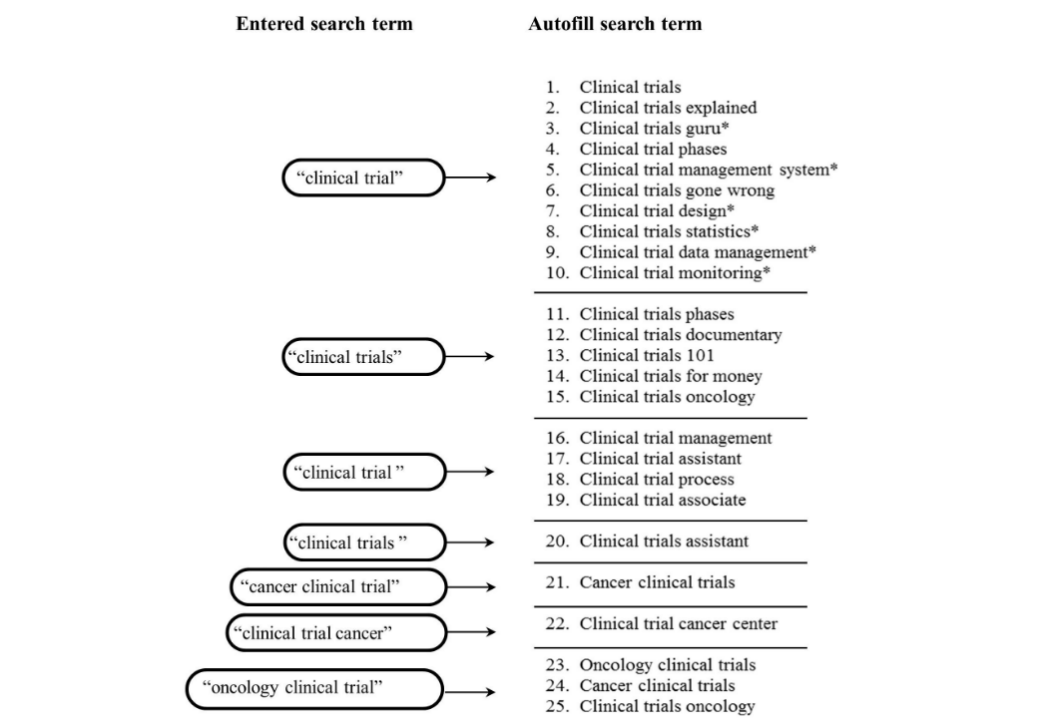
Keywords and search terms. Asterisk indicates terms considered irrelevant and excluded.

YouTube videos posted prior to March 2017 were searched for using the keywords “clinical trial(s)” (426,000 videos), “cancer clinical trial(s)” (352,000 videos), “clinical trials cancer” (611,000 videos), and “oncology clinical trial” (619,000 videos). To reduce bias introduced in the display of videos by the search engine due to the location and search history of the study computer, searches were conducted using the incognito mode of Google Chrome in a single day, with results captured for later assessment [26]. A snowballing technique was used to select videos for review wherein sequential screening of the autofill search results for each set of keywords was conducted. A total of 25 search term options were initially identified (Figure 1); 6 search term options were deemed irrelevant and excluded. The first 30 videos from each of the 19 remaining search term options were recorded; duplicate videos were removed, yielding 291 cancer clinical trial videos. YouTube uses a complex algorithm to rank video quality that is based on the duration the video has been watched. Longer viewer time indicates that the video is most likely appropriate for the search terms employed, which results in a higher ranking and greater likelihood of the video appearing on top of a search list [28].

Videos with <200 views (n=77) and those deemed irrelevant (n=80) were removed. Of the remaining 134 videos, additional 19 videos were found to be irrelevant upon viewing and were excluded from the final analysis. The remaining 115 videos were reviewed by 4 independent reviewers (GCH, SAM, KMS, and MB). Interrater reliability of the video characteristics and content-related variables, excluding the number of views, video length, and “thumbs up” and “thumbs down” was assessed by a fifth reviewer (CHB) and was found to be high among a randomly selected 10% sample (Cohen kappa=0.85).

The following video characteristics were assessed: number of views, length of the video in minutes, year published, country of origin, video source (nonprofit organization, health care provider/organization or cancer center, school/educational organization, pharmaceutical or biotech company, clinical research organization, or other), and YouTube video category (nonprofits and activism, science and technology, education, people and blogs, or other). Style of communication was categorized as affective versus cognitive.

Videos were coded as affective in style if the content was presented verbally with overt positive or negative emotion that was persuasive in nature, whereas videos in which the content was delivered nonpersuasively, verbally or nonverbally, and without overt emotion were coded as cognitive in style. Also evaluated was the tone of the video (positive, negative, or neutral). Themes arising in the video (altruism/positive emotions, risks and dangers of clinical trials, advancing science, importance of volunteering for clinical trials, and other), the intended audience (patients, general public, caregivers, health care professionals, students, and research personnel assessed as to whom the information was being addressed), and the manner of presentation (lecture/course format/webinar, interview, testimonial, advertisement for paid participation, and other) were also evaluated. Viewer perceptions concerning the videos were also captured by assessing the “thumbs up,” “thumbs down,” and comment counts. Using the National Cancer Institute (NCI) Clinical Trials Information for Patients and Caregivers [29] series of documents as the reference standard, the content of each video was grouped *a priori* into five broad categories: clinical trial purpose, trial phases, study design, safety and ethics, and participant considerations. Reviewers derived the themes embedded in the content throughout the video viewing process; categorization of themes (eg, altruism/positive emotions, risks/dangers, advancing science, importance of volunteering, and other) was based on reviewer consensus.

Descriptive analyses, including calculation of frequency distributions, means (SD), and ranges, were performed. To assess video characteristics and content differences between videos for cancer clinical trials and clinical trials in general or videos with a focus on a disease other than cancer, univariable analyses using chi-square test for categorical variables and analysis of variance for continuous variables were conducted. Stepwise multivariable logistic regression models, controlling for the year of publication, were constructed to assess the associations between video type (cancer vs noncancer) and variables with *P* values <.05 in the univariable analysis, controlling for the year of upload. All analyses were performed using IBM SPSS (version 24) [30]. Institutional review boards of each author’s respective institution deemed nonhuman subject research exempted from review.

## Results

The 115 videos related to clinical trials were viewed by approximately 11 million viewers with a mean number of 94,360 (SD 827,883) views each (Table 1).

**Table 1:** Characteristics of YouTube videos related to clinical trials.

Variable			Total (n=115)		Cancer related (n=46)		Noncancer related (n=69)	*P* value
**Number of views**			10,851,366		1,475,392		9,375,974	
	Mean (SD)		94,359.7 (827,883.4)		32,073.7 (180,025.0)		135,883.7 (1,059,819.2)	.20
	Range		216-8,810,958		226-1,223,520		216-8,810,958	
**Length of video (minutes)**								
	Mean (SD)		7.95 (11.2)		6.6 (6.9)		8.8 (13.3)	.03
	Range		0.57-61.0		0.8-35.9		0.6-61.0	
**Year published, n (%)**								.48
	2005-2010		16 (13.9)		9 (19.6)		7 (10.1)	
	2011-2012		29 (25.2)		10 (21.7)		19 (27.5)	
	2013-2014		39 (33.9)		14 (30.4)		25 (36.2)	
	2015-2016		31 (27.0)		13 (28.3)		18 (26.1)	
**Country of origin, n (%)**								.09
	United States		94 (81.7)		41 (89.1)		53 (76.8)	
	Other		21 (18.3)		5 (10.9)		16 (23.2)	
**Video source, n (%)**								<.001
	Nonprofit organization		22 (19.1)		10 (21.7)		12 (17.4)	
	Health care organization/cancer center		34 (29.6)		23 (50.0)		11 (15.9)	
	School/educational organization		7 (6.1)		0 (0.0)		7 (10.1)	
	Pharma/biotech		8 (7.0)		1 (2.2)		7 (10.1)	
	CRO^a^/recruitment agency		19 (16.5)		3 (6.5)		16 (23.2)	
	Other		25 (21.7)		9 (19.6)		16 (23.2)	
**YouTube category, n (%)**								.47
	Nonprofits & activism		24 (20.9)		13 (28.3)		11 (15.9)	
	Science & technology		41 (35.7)		14 (30.4)		27 (39.1)	
	Education		30 (26.1)		10 (21.7)		20 (29.0)	
	People & blogs		10 (8.7)		5 (10.9)		5 (7.2)	
	Other		10 (8.7)		4 (8.7)		6 (8.7)	
**Communication style, n (%)**								
	Affective		49 (42.6)		25 (54.3)		24 (34.8)	.04
	Cognitive		79 (68.7)		29 (63.0)		50 (72.5)	.29
**Tone, n (%)**								.04
	Positive		74 (64.3)		35 (76.1)		39 (56.5)	
	Negative		9 (7.8)		4 (8.7)		5 (7.2)	
	Neutral		32 (27.8)		7 (15.2)		25 (36.2)	
**Theme, n (%)**								
	Altruism/positive emotions		31 (27.0)		18 (39.1)		13 (18.8)	.016
	Risks/dangers		11 (9.6)		4 (8.7)		7 (10.1)	1.00
	Advancing science		9 (7.8)		5 (10.9)		4 (5.8)	.48
	Importance of volunteering		13 (11.3)		6 (13.0)		7 (10.1)	.63
	Other		9 (7.8)		2 (4.3)		7 (10.1)	.31
**Intended Audience, n (%)**								
	Patients		67 (58.3)		37 (80.4)		30 (43.5)	<.001
	General public		68 (59.1)		25 (54.3)		43 (62.3)	.39
	Caregivers		1 (0.9)		1 (2.2)		0 (0.0)	.56
	Health care professionals		13 (11.3)		4 (8.7)		9 (13.0)	.47
	Students		6 (5.2)		0 (0.0)		6 (8.7)	.08
	Research personnel		6 (5.2)		1 (2.2)		5 (7.2)	.40
**Presentation, n (%)**								
	Lecture/course/webinar		69 (60.0)	26 (56.5)			43 (62.3)	.53
	Interview		16 (13.9)	6 (13.0)			10 (14.5)	.83
	Testimonial		31 (27.0)	17 (37.0)			14 (20.3)	.048
	Advertisement		6 (5.2)	4 (8.7)			2 (2.9)	.22
	Other		21 (18.3)	7 (15.2)			(20.3)	.49
**Viewer Perceptions**								
	**Thumbs up**							
		Mean (SD)	963.7 (9920.3)	33.3 (132.9)			1584.0 (12806.1)	.11
		Range	0-10,6415	0-897			0-10,6415	
	**Thumbs down**							
		Mean (SD)	285.1 (2963.3)	18.0 (116.1)			463.01 (3825.2)	.11
		Range	0-31,777	0-788			0-31,777	
	**Comments**							
		Mean (SD)	99.4 (1009.4)			1.5 (3.4)	164.6 (1302.9)	.10
		Range	0-10,824			0-15	0-10,824	

^a^CRO: clinical research organization.

Forty-six of 115 (40.0%) videos discussed cancer clinical trials versus 69/115 (60.0%) that either focused on other diseases (eg, Parkinson’s disease) or were general discussions of clinical trials, not related to any specific disease. The mean length of a video was approximately 8 min (SD 11.2) and the majority (94/115, 81.7%) of the videos were produced in the United States. Videos created by health care organizations, including cancer centers, predominated (34/115, 29.6%), and many were posted under the “science and technology” theme of YouTube.

Overall, videos were oriented toward patients (67/115, 58.3%) and the general public (68/115, 59.1%), tended to be cognitive (79/115, 68.7%) in nature, and were presented as a lecture/course/webinar (69/115, 60%). The most popular theme among clinical trial videos was altruism and other positive emotions associated with clinical trial participation (31/115, 27.0%) followed by the importance of volunteering for trials (13/115, 11.3%).

Compared with noncancer-related videos, cancer clinical trial videos were shorter in length (6.6 vs 8.8 min, *P*=.03) and were more often created by health care organizations (23/46, 50.0% vs 11/69, 15.9%, *P*<.001). More than half of the cancer videos used an affective approach compared to about one-third of noncancer videos (*P*=.04) and, compared to noncancer videos, more often expressed a positive tone (35/46, 76.1% vs 39/69, 56.5%, *P*=.04) toward clinical trials and focused on altruism and other positive emotions (18/46, 39.1% vs 13/69, 18.8%, *P*=.016). Compared with only 43.5% (30/69) of noncancer videos (*P*<.001), 80% (37/46) of cancer videos were geared toward a patient population and were most often presented as testimonials (patient and physician; 37% (17/46) vs 20.3% (14/69), *P=*.048).

With regard to the content, the most commonly mentioned item was the purpose of a clinical trial (62/115, 53.9%), followed by the fact that clinical trials are conducted in phases (44/115, 38.3%), information about specific phases (Phase I=46/115, 40.0%, Phase II=37/115, 32.2%, and Phase III=40/115, 34.8%), there is eligibility criteria for entering a trial (37/115, 32.2%), and that there are benefits to participating in a clinical trial (38/115, 33.0%; Table 2). Cancer-related clinical trial videos more often mentioned that Phase I studies evaluate how the drug effects the body and are used to determine potential side effects (4/46, 8.7% vs 0/69, 0%, *P*=.02) and that they may be offered in cases when no standard treatment options exist (5/46, 10.9% vs 0/69, 0%, *P*=.009) compared with noncancer-related videos. Cancer-related videos also differed from noncancer-related videos in that cancer-related videos more frequently mentioned the benefits of clinical trial participation, such as better care and monitoring (21/46, 45.7% vs 11/69, 15.9%, *P*<.001), participants could be the first to benefit from an experimental treatment if it works (19/46, 41.3% vs 7/69, 10.1%, *P*<.001), and their participation could very well help others in the future (20/46, 43.5% vs 13/69, 18.8%, *P*=.004). 

**Table 2:** Clinical trial YouTube video content.

Variable			Total (n=115), n (%)		Cancer related (n=46), n (%)		Noncancer related (n=69), n (%)		*P* value	
**Purpose**										
	Mentions purpose in general		62 (53.9)		28 (60.9)		34 (49.3)		.22	
	Test new drugs/devices in human subjects		20 (17.4)		10 (21.7)		10 (14.5)		.32	
	Determine a safe drug dose		10 (8.7)		2 (4.3)		8 (11.6)		.31	
	Determine drug efficacy		5 (4.3)		1 (2.2)		4 (5.8)		.65	
	Test a research question		3 (2.6)		1 (2.2)		2 (2.9)		1.00	
**Trial phases**										
	Mentions there are phases in general		44 (38.3)		16 (34.8)		28 (40.6)		.53	
	Phase I		46 (40.0)		18 (39.1)		28 (40.6)		.88	
	Determine dosing		33 (28.7)		13 (28.3)		20 (29.0)		.93	
	Assess safety		15 (13.0)		9 (19.6)		6 (8.7)		.09	
	Determine method of administration		15 (13.0)		5 (10.9)		10 (14.5)		.57	
	Small sample size		24 (20.9)		6 (13.0)		18 (23.1)		.09	
	Healthy volunteers		6 (5.2)		2 (4.3)		4 (5.8)		1.00	
	Compensation for participation		3 (2.6)		1 (2.2)		2 (2.9)		1.00	
	How the drug affects the body/side effects		4 (3.5)		4 (8.7)		0 (0.0)		.02	
	When no other standard treatment options are available		5 (4.3)		5 (10.9)		0 (0.0)		.009	
	Phase II		37 (32.2)		15 (32.6)		22 (31.9)		.93	
	Determine effect on disease course		31 (27.0)		13 (28.3)		18 (26.1)		.80	
	<100 sample size		20 (17.4)		4 (8.7)		16 (23.2)		.045	
	Phase III		40 (34.8)		17 (37.0)		23 (33.3)		.69	
	Compare to standard treatment		32 (27.8)		16 (34.8)		16 (23.2)		.17	
	>100 sample size		24 (20.9)		7 (15.2)		17 (24.6)		.22	
	Phase IV		13 (11.3)		3 (6.5)		10 (14.5)		.19	
	Postmarketing testing for side effects		8 (7.0)		3 (6.5)		5 (7.2)		1.00	
**Study design**										
	Randomized controlled trial		29 (25.2)		13 (28.3)		16 (23.2)		.54	
	Reduce bias		14 (12.2)		5 (10.9)		9 (13.0)		.73	
	Control group		24 (20.9)		12 (26.1)		12 (17.4)		.26	
	Interventional group		24 (20.9)		10 (21.7)		14 (20.3)		.85	
	Blinding		16 (13.9)		5 (10.9)		11 (15.9)		.44	
	Placebo trial		25 (21.7)		8 (17.4)		17 (24.6)		.36	
	Research team		33 (28.7)		16 (34.8)		17 (24.6)		.24	
**Safety and ethics**										
	FDA^a^ regulatory process		9 (7.8)		4 (8.7)		5 (7.2)		1.00	
	Written protocols/strict guidelines		12 (10.4)		4 (8.7)		8 (11.6)		.76	
	Eligibility criteria		37 (32.2)		17 (37.0)		20 (29.0)		.37	
	Protection of safety		30 (26.1)		10 (21.7)		20 (29.0)		.39	
	IRB^b^		12 (10.4)		4 (8.7)		8 (11.6)		.76	
	DSMB^c^		5 (4.3)		3 (6.5)		2 (2.9)		.39	
	FDA		19 (16.5)		8 (17.4)		11 (15.9)		.84	
	Ethical conduct of research		4 (3.5)		2 (4.3)		2 (2.9)		1.00	
	Informed consent		34 (29.6)		13 (28.3)		21 (30.4)		.80	
	Explanation of purpose, procedures, benefits, and harms		29 (25.2)		14 (30.4)		15 (21.7)		.29	
	Voluntary nature of participation		31 (27.0)		16 (34.8)		15 (21.7)		.12	
	Ability to withdraw at any time		20 (17.4)		11 (23.9)		9 (13.0)		.13	
**Participant considerations**										
	**Potential benefits**									
		Mentions benefits in general		38 (33.0)		19 (41.3)		19 (27.5)		.12
		Better care and monitoring		32 (27.8)		21 (45.7)		11 (15.9)		<.001
		First to benefit if treatment works		26 (22.6)		19 (41.3)		7 (10.1)		<.001
		Help others in the future		33 (28.7)		20 (43.5)		13 (18.8)		.004
	**Potential risks**									
		Mentions risks in general		29 (25.2)		10 (21.7)		19 (27.5)		.48
		Not always better than standard treatment		4 (3.5)		2 (4.3)		2 (2.9)		1.00
		No guarantee of effectiveness		19 (16.5)		9 (19.6)		10 (14.5)		.47
		Unknown side effects		18 (15.7)		5 (10.9)		13 (18.8)		.25
		Costs associated with participation		16 (13.9)		12 (26.1)		4 (5.8)		.002
		Communication with physician		32 (27.8)		24 (52.2)		8 (11.6)		<.001
		Communication with family		11 (9.6)		8 (17.4)		3 (4.3)		.03
		Quality of life		16 (13.9)		11 (23.9)		5 (7.2)		.01

^a^FDA: Food and Drug Administration.

^b^IRB: institutional review board.

^c^DSMB: Data Safety Monitoring Board.

Additionally, the cost associated with participation (12/46, 26.1% vs 4/69, 5.8%, *P*=.002), the importance of communication with one’s doctor (24/46, 52.2% vs 8/69, 11.6%, *P*<.001) and family (8/46, 17.4% vs 3/69, 4.3%, *P*=.03), and the quality of life (11/46, 23.9% vs 5/69, 7.2%, *P*=.01) were all mentioned more often in cancer-related videos than in noncancer-related videos.

Results of the multivariable regression analysis demonstrated that compared with noncancer clinical trial videos, videos related to cancer clinical trials are much more likely to have been created by health care organizations, including cancer centers (odds ratio [OR] 5.95, 95% CI 1.70-20.88), to mention the costs associated with clinical trial participation (OR 5.93, 95% CI 1.15-29.46) and to advise patients to communicate with their physician about cancer clinical trials (OR 4.94, 95% CI 1.39-17.56; Table 3).

**Table 3:** Video characteristics and content associated with cancer clinical trial YouTube videos.

Variable		Odds ratio (95% CI)		*P* value	
**Year published**					
	2005-2012	Reference			
	2013-2016	1.87 (0.61-5.70)		.27	
**Video source**					
	Other (school, CRO^a^, education, other)	Reference			
	Health care/cancer center	5.95 (1.70-20.88)		.005	
**Communication style**					
	Affective	0.63 (0.18-2.18)		.47	
**Tone**					
	Negative	Reference			
	Positive	3.78 (0.41-35.20)		.24	
	Neutral	1.45 (0.31-6.77)		.63	
**Theme**					
	Altruism/positive emotions	2.26(0.55-9.34)			.26
**Intended audience**					
	General public	2.25 (0.71-7.12)			.17
**Participant considerations**					
	Potential benefits	—			—
	Better care and monitoring	2.30 (0.63-8.41)			.21
	First to benefit if treatment works	2.19 (0.65-7.41)			.21
Costs associated with participation		5.83 (1.15-29.46)			.033
Communication with physician		4.94 (1.39-17.56)			.013
Communication with family		1.03 (0.14-7.63)			.98
Quality of life		2.15 (0.50-9.20)			.30

^a^CRO: clinical research organization.

## Discussion

### Principal Findings

Our review of the 115 top viewed YouTube videos revealed that a large proportion of these videos are devoted to cancer clinical trials. Overall, clinical trial videos convey information that is aimed at both patients and the general population audiences. The majority of the videos presented the viewer with the overall purpose of a clinical trial and many discussed the phases of clinical trials and the fact that criteria are used to determine a patient’s eligibility for enrollment. Beyond these topics, the video content varied widely, with most touching upon selected topics (eg, phases of clinical trials, federal regulations, informed consent or benefits of enrollment, and the importance of communication with a physician). Interestingly, none discussed the concept of clinical equipoise.

Cancer clinical trial videos were more positive in tone and more frequently used an affective communication style. They tended to emphasize altruism, the importance of volunteering to participate in a trial, and the benefits of participation more so than did non-cancer videos. Further, cancer clinical trial videos were nearly six times as likely to be created by a health care organization or a cancer center and were much more likely than noncancer trial videos to communicate practical information about clinical trial participation costs and to encourage dialogue with one’s physician.

### Limitations

Much attention was taken in the selection of the videos reviewed in this study to represent the most commonly viewed YouTube videos about clinical trials however, selecting the top 30 videos with greater than 200 views may have introduced a selection bias. Since the YouTube video ranking algorithm places videos with longer user viewing times at the top of the list and overlap was found in the videos in the top 30 for the search terms, our inclusion criteria likely captured the most widely viewed YouTube videos related to clinical trials. Further, we postulated that any bias introduced by the algorithm would similarly influence the videos displayed when a consumer uses the same search term and that the impact of less-viewed videos would be minimal. Despite using search terms specific to cancer clinical trials, we found that a large proportion of clinical trial videos were not related to cancer. While this was an unexpected finding, a consumer using our search terms would likely have the same experience. Whether or not viewers were engaged for the full duration of any video is unknown as 30 seconds of YouTube watching is considered a “viewing” [31]. This study is also limited in that it was cross-sectional in design and is further compounded by the fact that new videos are continually being uploaded on YouTube. Finally, this study focused solely on English language videos.

### Comparison With Prior Work

To date, no other study has evaluated the contents of YouTube videos regarding clinical trials. More than 800 peer-reviewed publications reporting on the quality and content of YouTube videos relating to public health topics ranging from anorexia [32] to Zika virus [33] now exist, which is cause for concern regarding the power of this medium to communicate information accurately and responsibly to the general public. The decision to take part in a cancer clinical trial is a complex one, and the most common barrier to participation is lack of knowledge about cancer clinical trials [33]. Two separate studies found that the internet and media are the primary sources for learning about clinical trials [34] and that the information “read, saw, or heard” about a study was a major influence on the decision to participate. Further evidence supports that seeking information about one’s illness can be viewed as a key coping strategy, which may lead to health-promotive activity and facilitate psychosocial adjustment to illness [35]. Use of the internet as a source of health-related information, however, has been likened to drinking from a fire hose and not knowing the source of the water [36], a sentiment that can easily be applied to YouTube video viewing. Because there exists no arbiter of the truth or accuracy of the material posted on YouTube, many question both the credibility and accuracy of the information and find that the content is influenced by perspectives of the video source [37-42]. Currently, the NCI acknowledges the importance of social media as a source of health-related information, and through its Cancer Moonshot Initiative, seeks to leverage this platform to provide patients with reliable information by developing a social media best practices toolkit. Information learned in this study showed that the majority of the clinical trial information communicated was accurate, as determined using NCI information for comparison [29] and was conveyed in a positive and compassionate manner. The coverage of topics however, was spotty, and the sufficiency and quality of information was lacking many times.

### Conclusions

Overall, YouTube clinical trial videos provided information on many aspects of clinical trials, particularly cancer clinical trials. Few covered the full range of concepts needed to make an informed decision about participation; the majority focused on selected topics and provided varying levels of detail, leaving the viewer with an incomplete view of key concepts and partially informed. Given the abundance of clinical trial videos and relative ease of access to this information, care must be taken by patients and their families to verify and supplement YouTube video information with consultations with their healthcare professional to obtain a full and accurate picture of cancer clinical trials, thus, to make an adequately informed decision about participation.

## Abbreviations

CRO: clinical research organization
DSMB: Data Safety Monitoring Board
FDA: Food and Drug Administration
NCI: National Cancer Institute
OR: odds ratio
